# Beneficial Outcome Pathways: A New Mechanistic Framework to Map the Health Effects of Fasting in Metabolic Dysfunction‐Associated Steatotic Liver Disease

**DOI:** 10.1155/jnme/3190642

**Published:** 2026-04-09

**Authors:** Robin Mesnage, Verena Buchinger

**Affiliations:** ^1^ Buchinger Wilhelmi Clinic, Wilhelm-Beck-Straße 27, Überlingen, 88662, Germany; ^2^ Department of Nutritional Sciences, School of Life Course Sciences, Faculty of Life Sciences and Medicine, King’s College London, London, SE1 9NH, UK, kcl.ac.uk

**Keywords:** AMPK, autophagy, beneficial outcome pathways, causal inference, fasting, ketosis, lifestyle medicine, MASLD

## Abstract

Lifestyle interventions such as fasting are difficult to evaluate using placebo‐controlled randomized trials, resulting in fragmented and heterogeneous evidence. The beneficial outcome pathways (BOPs) framework can address this limitation by integrating mechanistic data to clarify the causal processes leading to beneficial health outcomes, adapted from the adverse outcome pathway concept in toxicology. To illustrate the approach, we apply the BOP framework to fasting interventions for metabolic dysfunction‐associated steatotic liver disease (MASLD). The initial trigger is an acute energy deficit that activates AMP‐activated protein kinase (AMPK) signalling and inhibits mTORC1. Downstream key changes include the activation of autophagy and lipophagy, the suppression of de novo lipogenesis, the enhancement of fatty acid oxidation and ketogenesis, anti‐inflammatory changes, and the restoration of hepatic and systemic insulin sensitivity. Overall, this new framework helps connect laboratory findings with clinical results and provides a clearer way to understand how fasting improves MASLD.

## 1. Introduction

A major methodological challenge in evaluating health‐promoting interventions is that they cannot always be tested using gold‐standard randomized controlled trials (RCTs) with placebos and tightly matched control groups [1, 2]. This limitation is particularly evident in complex lifestyle domains such as for whole diet nutritional interventions [3], behavioural modifications [4], sleep optimization [5] or exercise interventions [6]. Consequently, the evidence supporting the effectiveness of these interventions often appears fragmented, heterogeneous or “less rigorous” than that for pharmaceuticals.

**FIGURE 1:**
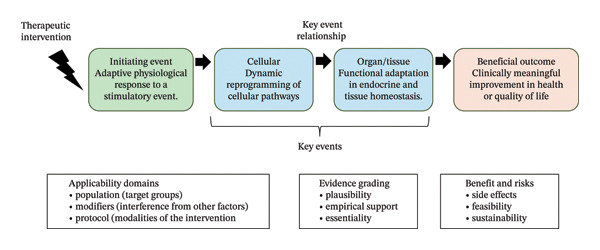
Architecture of beneficial outcome pathways.

Beneficial outcome pathways (BOPs) framework can address this limitation by integrating mechanistic data to clarify the causal processes leading to beneficial health outcomes (Figure 1).

The design of BOPs is inspired by the adverse outcome pathway (AOP) framework originally developed in toxicology [7]. By structuring mechanistic knowledge through a series of key events (KEs) leading to an adverse outcome, AOPs have been useful to determine the toxicity of chemicals which would be unethical to test directly on humans as well as to minimise the use of animals in toxicity tests. This includes, but is not limited to, acetylcholinesterase inhibition leading to neurotoxicity [8], chronic estrogen receptor activation causing uterine adenocarcinoma [9], bile‐salt export pump inhibition resulting in cholestatic liver injury [10] and mitochondrial β‐oxidation inhibition driving hepatic steatosis [11]. The AOP framework has also been applied to organise mechanistic data in COVID‐19 research outlining plausible sequences from SARS‐CoV‐2 infection and ACE2 receptor binding to IL‐6 secretion, systemic inflammation, and ultimately respiratory distress [12].

BOPs complement, rather than replace, RCTs. They provide a framework for mechanistic integration when trials are impractical or confounded. By prespecifying proximal KEs and mediators, BOPs allow smaller studies to test parts of the causal chain, strengthening inference without relying solely on long‐term clinical outcomes.

## 2. Architecture of BOPs

The architecture of BOPs parallels the established structure of AOPs (Table 1).

**TABLE 1:** Core components of a beneficial outcome pathway (BOP).

Component	Definition
Initiating Event (IE)	The first measurable perturbation caused by the intervention
Key Events (KEs)	Measurable biological changes at molecular, cellular, tissue or systemic levels
Key Event Relationships (KERs)	Causal links between IEs and KEs or between successive KEs
Beneficial Outcome (BO)	Clinically meaningful improvement in health or quality of life
Applicability Domain	Context in which the BOP is valid (population, protocol and modifiers)
Evidence Grading	Confidence in each node and relationship, based on plausibility, empirical support, and essentiality

A BOP begins with an molecular initiating event (MIE), defined as a measurable and reproducible perturbation induced by an intervention. This initiating state sets in motion a cascade of biological responses that can be systematically described as a sequence of which represent measurable changes at different levels of biological organization, ranging from molecular signalling to tissue remodelling and systemic physiology. The causal links between events, termed KE relationships (KERs), form the backbone of a BOP. These relationships require explicit consideration of their biological plausibility, empirical support and essentiality, criteria adapted from OECD guidance for systematic AOP evidence grading [13, 14]. A simplified three‐tier scale (high, moderate and low confidence) facilitates the assessment of the strength of evidence.

The BO represents a clinically meaningful improvement. While changes in validated biomarkers or risk factors, such as blood pressure, LDL cholesterol or tumour size, can act as surrogate indicators of therapeutic efficacy, they qualify as BOs only when they translate into tangible improvements in how a person feels, functions or survives [15]. Extending beyond purely biological measures, BOs encompass intermediate outcomes that capture functional or symptomatic relief, such as reduced angina frequency, improved exercise tolerance or enhanced vitality. Thus, for instance, although hypertension is formally a risk factor for stroke, its alleviation can be considered a BO when it leads to perceptible gains in quality of life [16].

Importantly, a BOP does not require every patient to achieve the BO; rather, it provides a mechanistic map of how the intervention can plausibly lead to that outcome in at least a subset of individuals. Because lifestyle interventions are highly context‐dependent, each BOP must also specify its applicability domain. This includes the target population (for example, individuals with metabolic syndrome versus healthy volunteers) and potential modulating factors such as age, sex, baseline adiposity, medication use and comorbidities which are known to alter molecular and physiological responses to nutritional or behavioural interventions [16].

## 3. Strengthening the Evidence Base for the Therapeutic Effects of Fasting in Metabolic Dysfunction‐Associated Steatotic Liver Disease (MASLD)

We explore in the next sections how BOP can be applied to better understand and document the health effects of fasting interventions in fatty liver diseases [17–21]. Nonalcoholic fatty liver disease (NAFLD), recently redefined as MASLD, arises from the combined effects of chronic energy surplus, hepatic de novo lipogenesis, impaired fat oxidation, insulin resistance, and low‐grade inflammation [22]. MASLD is a multifactorial and a multistage disease [23]. MASLD progresses through four histological stages: simple steatosis, steatohepatitis (MASH), fibrosis and cirrhosis. The transition from steatosis to steatohepatitis marks the key turning point toward progressive disease. Fibrosis severity increases with higher inflammation and hepatocellular injury.

Switching from the typical western diet, characterised by excessive intake of processed foods, red and processed meats, high‐fat dairy, refined grains, and sugar, while being low in fruits, vegetables and fiber, to a Mediterranean diet or any dietary variations involving a more healthy dietary pattern has demonstrated efficacy in MASLD treatment [24]. It is not clear whether benefits are due to changes in the quantity or the quality of the food, or even to the time at which the food is eaten. Many clinical studies where a switch to a healthy dietary pattern causes a positive health outcome are linked to a reduction in energy intake leading to weight loss [24].

Fasting strategies including intermittent fasting (IF) (from time‐restricted eating [TRE] to 5:2 regimens) and long‐term fasting (LTF) are increasingly recognised as a therapy to address MASLD [17–21]. In a meta‐analysis of 21 studies involving 1226 participants, fasting effectively decreased liver fat and steatosis, independent of the participant age, health status, weight status or intervention duration [25, 26]. However, it remains unclear which fasting interventions are most effective at each stage of MASLD.

Among all fasting strategies, IF protocols have been the most extensively studied. In an 8‐week randomised trial of modified ADF in MASLD patients, the IF group achieved significant weight loss (∼5% body weight) and reductions in liver enzymes and fibrosis markers compared to a no‐diet control [27]. A longer 12‐week trial in China compared a 5:2 diet against continuous calorie restriction in 60 MASLD patients. Both groups lost similar weight, but the 5:2 regimen led to greater liver‐specific benefits: the prevalence of moderate‐to‐severe steatosis dropped to 30% in the 5:2 diet group versus 59% in the daily restriction group, and fewer 5:2 diet patients had significant fibrosis [28].

TRE, limiting food intake to a shortened daily window (e.g., 6–10 h) without necessarily cutting total calories, is another popular fasting paradigm. In the landmark TREATY‐FLD trial, 88 patients with obesity and MASLD were randomised to 8‐h TRE versus standard daily calorie restriction. After 12 months, both groups achieved ∼8% absolute reductions in liver fat, and ∼40% of participants in each arm had resolution of fatty liver. Crucially, TRE provided no additional liver fat reduction beyond caloric restriction which suggested that in the case of TRE the benefits are mediated by the weight loss and not fasting per se [29]. Whether individuals practise TRE early or late during the day can also have an influence. In a network meta‐analysis of 12 RCTs including 730 adults with overweight or obesity, early and late time‐restricted eating produced similar moderate weight loss, but early TRE showed greater improvements in insulin resistance and some metabolic outcomes compared with late TRE [30].

These findings imply that aligning feeding to the body’s circadian rhythm (as TRE does) might enhance certain hormonal and autophagy‐mediated mechanisms that benefit the liver in some cases, even if overall fat loss is similar. From a mechanistic standpoint, TRE produces some of the fasting‐state metabolic shifts observed in longer fasting. During the daily fasting interval, hepatic glycogen is depleted and insulin levels fall, but whether this is sufficient to prompt increased lipolysis in adipose tissue to an extent which is efficient to trigger further health effects remains debatable.

Fasting seems to be also beneficial in MASH patients. However, the results of trials are more heterogenous with fasting protocols being effective in addressing fibrosis only in obese patients [25]. Recent preclinical data suggest that an IF 5:2 regimen can prevent the development of MASH, ameliorate established fibrosis, and blunt the transition from MASH to hepatocellular carcinoma through cooperative activation of PPARα‐ and PCK1‐mediated metabolic pathways [31]. These findings also indicate that fasting may exert hepatoprotective effects independent of caloric restriction.

There are some limitations. Fasting interventions may also have detrimental effects on muscle mass in patients with liver cirrhosis who are prone to the catabolic consequences of fasting due to anabolic resistance [32]. Severe disruption of hepatic function (e.g., decompensated cirrhosis) can also alter the liver’s ability to maintain glycaemic control, which can be a risk of adverse effects during fasting [33].

The case of Ramadan as a religious fast is particularly compelling to examine, as it is practised by billions of people across regions where visceral adiposity and Type 2 diabetes are spreading with alarming speed. In a 2011 multicentre study of 300 cirrhotic patients fasting during Ramadan, several patients developed complications including fatigue, variceal bleeding, encephalopathy, and there were three deaths from hepatorenal syndrome. While mild cases (severity of chronic liver disease, Child A) showed modest metabolic improvements, severe cases faced significant risks [34]. Another study came to the similar conclusion that fasting is not contraindicated for cirrhotic patients with Child class A but that patients with Child class C should not fast [35]. A recent review of this data suggested that fasting should be avoided in patients with active peptic ulcers, while those with inactive ulcers should receive treatment and continue PPI therapy during fasting [36].

## 4. Energy Deficit as the Initiating Event of Fasting‐Induced Therapeutic Mechanisms

The MIE of fasting is the acute energy deficit created by the withdrawal of external nutrient supply [37, 38]. When glucose levels are dropping because glycogen stores are exhausted, intracellular ATP production is lowered in several organs, setting the stage for activation of the cellular energy sensor AMPK. Rising AMP/ATP and ADP/ATP ratios allosterically activate AMP‐activated protein kinase (AMPK). Once active, AMPK functions as a metabolic switch, inhibiting ATP‐consuming processes such as lipid and protein synthesis, while stimulating energy‐provision pathways including fatty acid oxidation, glucose uptake, and autophagy.

### 4.1. How Fasting Protocols Differ in Their Potential to Trigger the MIE

The characteristics of the fasting intervention itself define whether the MIE is triggered. Fasting protocols vary widely in their duration, degree of caloric restriction, and macronutrient profile, factors that determine both the metabolic switch and downstream adaptive responses. According to the 2024 International Consensus on Fasting Terminology [39], fasting can be broadly classified into several categories:

• Short‐term fasting (STF) lasting 2–3 days, typically fluid‐only.
• Prolonged or LTF (PF/LTF) lasting ≥ 4 days, often applied therapeutically (e.g., Buchinger fasting).
• IF represented by repetitive fasting periods lasting ≤ 48 h, including alternate‐day fasting (ADF) and alternate‐day modified fasting (ADMF), in which eating and fasting alternate across days.
• TRE as daily restriction of food intake to a consistent 6–10 h eating window, resulting in a 14–18 h fasting window, typically without an explicit reduction in total caloric intake.
• Fasting‐mimicking diets (FMD) which are ∼1000 kcal/day regimens designed to mimic the metabolic effects of fasting without complete abstinence from food.

Each of these fasting models engages the fasting response to varying degrees, depending on the length of the fasting window, macronutrient composition, and extent of caloric restriction.

The optimal balance between kcal amount and timing of fasting for humans is still being researched. The literature is indeed less abundant on some specific biomarkers (e.g., exact autophagy quantification in humans) but is growing. In a study on human skeletal muscle, a 36‐h fast was found to clearly modulate AMPK–mTOR signalling and several autophagy mediators, whereas shorter fasting periods produced only modest and ambiguous changes in autophagy markers [40]. Another hallmark of the fasting metabolic switch, growth hormone (GH), rises sharply after 24–48 h of fasting, a response that appears largely independent of weight loss. GH secretion increased up to fivefold after 2 days of fasting, driven by more frequent and higher‐amplitude secretory bursts [41]. Similarly, 24 h water‐only fasting in healthy individuals markedly elevated GH levels, with the greatest relative increases observed in those starting with low basal GH [42].

The macronutrient profile matters. In an RCT, combining an 8 h TRE regimen with a low‐carbohydrate diet produced the most pronounced improvements in glycaemic control and dyslipidaemia [43]. Similarly, a recent trial demonstrated that FMDs both low‐protein/high‐fat (LP‐FMD) and high‐protein/low‐fat (HP‐FMD) formulations, improved cardiometabolic parameters and induced autophagy [44]. There were nonetheless differences, and HP‐FMD preferentially reduced visceral adiposity, improved lipid metabolism, and enhanced heart rate variability, whereas LP‐FMD induced greater ketogenesis and IGF‐1 suppression [44].

### 4.2. Practical Biomarkers of MIE Activation

Several hallmarks of the fasting metabolism can serve as biomarkers for the activation of the MIE. Ketonemia rapidly increase after 24 h of LTF [45]. In another study with water‐only fasting, ketonemia became detectable after 21.1 h without exercise and 17.5 h with exercise [46]. Ketonuria measures correlate well with the concentration in ß‐hydroxybutyrate measured in blood [45]. The nitroprusside test using Ketostix strips is known to be a reliable method to distinguish ketotic from nonketotic patients. We consider that activation of ketosis, which can be measured in urine with simple stripes, reflects well the transition to a catabolic state and is probably one of the most reliable and clinically practical biomarker for the activation of the MIE.

### 4.3. A Canonical Initiating Event

In order to facilitate classification, a canonical initiating event assuming substantial acute caloric withdrawal, typically ≤ 500 kcal/day, as in the definition of Buchinger fasting, which is one of the most well‐characterised fasting protocols, or complete caloric abstinence for over 24 h, can be defined [47]. In this case, although modifier apply, it can be expected that cellular energy stress will be sufficient to trigger the activation of the cascade of KE.

## 5. Applicability Domains and Modifiers of the MIE for Fasting Health Effects

Although the underlying biochemical logic of the MIE is universal, its amplitude, timing and safety margin vary according to the metabolic health, age, sex, and physiological reserve of the individual.

• *Age*: Older adults display slower AMPK activation and reduced mitochondrial adaptability [48], often accompanied by a higher proportion of proteolysis relative to lipid oxidation [49].
• *Sex*: Differences in weight loss have been reported in different studies, with men commonly losing more body weight and fat than women [50–52]. Men mobilise more abdominal fat than women who mostly use subcutaneous fat [53]. However, weight loss is likely to be equal regardless of sex when energy expenditure is equal [54].
• *Hormonal status*: Estrogen and androgens influence lipolytic sensitivity and hepatic fat oxidation [55]. Women and men can show different ketone kinetics and adipose mobilization during fasting [56], which may alter both the amplitude and timing of β‐HB rise and downstream anti‐inflammatory signalling.
• *Reproductive life stages*: Differences can become even more granular when looking at reproductive life stages. In premenopausal women undergoing a 60‐h fast, excessive adiposity modulated both insulin sensitivity and endocrine responses [57]. Indices of substrate utilization indicated that, despite a less pronounced metabolic adaptation to fasting, the overall metabolic flexibility of women with obesity remained comparable to that of lean counterparts, except in individuals presenting with metabolically unhealthy obesity.
• Recent food intake: Baseline glycogen status and recent diet can also have an influence. Persons having a chronically high‐carbohydrate, hyperinsulinemic state will have abundant hepatic glycogen and elevated de novo lipogenesis at baseline; they typically would show a more dramatic fall in insulin and a more pronounced switch to lipolysis once fasting begins [58]. Someone already mildly ketogenic (low‐carb habitual diet) may enter ketogenesis more rapidly [59].
• *Medications*: Sulfonylureas [60], SGLT2 inhibitors [61], glucocorticoids [62], beta‐blockers [63], and statins [64] all affect lipolysis, gluconeogenesis, or ketogenesis, even in the fed state. Those pharmacological backgrounds can modulate or even dominate the metabolic switch.
• *Physical activity and exercise*: Light aerobic activity accelerates glycogen depletion, increases AMP/ATP ratio in muscle, and augments whole‐body fatty acid oxidation, effectively advancing the timing of AMPK activation and ketone appearance [46, 65]. Sedentary fasting may therefore be slower to express the same MIE.
• *Diseases*: In decompensated states, including advanced hepatic fibrosis, severe cachexia, or insulin‐deficient diabetes, the same initiating event may not occur or may lead to maladaptive responses. Mitochondrial dysfunction in cirrhosis can blunt ketogenesis despite lipolysis [66], whereas uncontrolled type 1 diabetes causes unrestrained ketone accumulation and ketoacidosis [67].

In MASLD/MASH, mitochondrial dysfunction and hepatic insulin resistance may blunt fat oxidation and ketogenesis [68], particularly in more advanced disease stages. However, improvements in hepatic insulin signalling and lipid handling during IF may progressively restore the capacity for ketone production, in part through PPARα‐driven induction of fatty acid oxidation genes. This is supported by mouse studies showing that PPARα deficiency leads to hypoketonaemia and a markedly blunted transcriptional response to fasting [69].

Taken together, the fasting MIE is universally plausible but context‐dependent. It manifests with a highest reliability in adults who are metabolically unhealthy yet physiologically compensated: those capable of suppressing insulin, mobilising fat, and producing ketones without risking decompensation. Variability introduced by age, sex, baseline diet and co‐medication modifies the intensity and kinetics of the pathway but does not invalidate its causal logic. Explicit definition of these modifiers is therefore essential when applying the BOP framework to fasting interventions.

## 6. KEs and Mechanistic Sequence Leading to Resolution of MASLD by Fasting

**FIGURE 2:**
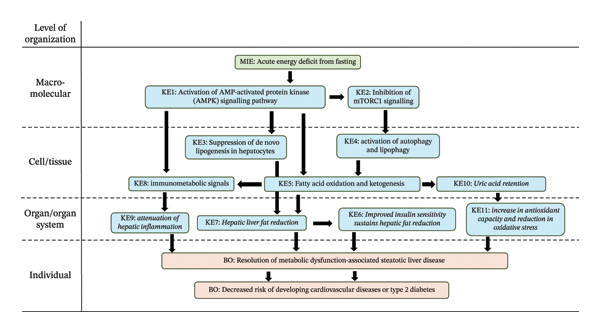
Resolution of metabolic dysfunction‐associated steatotic liver disease (MASLD) by fasting interventions. This schematic illustrates the mechanistic sequence through which fasting alleviates hepatic fat accumulation, framed using the logic of an adverse outcome pathway (AOP). The initiating event is an acute energy deficit triggered by fasting, which activates a series of mechanisms leading to fat elimination and normalisation of insulin tolerance, altogether creating a self‐reinforcing metabolic state that favours hepatic lipid clearance.

The resolution of MASLD by fasting interventions can be described as a mechanistic sequence (Figure 2).

Fasting initiates an acute energy deficit in hepatocytes (MIE), which raises the AMP/ATP ratio and activates the AMPK signalling pathway (KE1) [70]. Once switched on, AMPK acts as a metabolic master regulator, phosphorylating key enzymes to suppress energy‐consuming processes and promote energy‐producing ones. It inactivates acetyl‐CoA carboxylase, lowering malonyl‐CoA levels, which relieves inhibition of mitochondrial fatty acid import and stimulates β‐oxidation [71]. This AMPK‐driven metabolic shift (KE1) is well established as a prerequisite for lipid clearance, as pharmacological AMPK activation can normalize hepatic triglyceride content [72]. By contrast, AMPK inhibition is associated with the hepatic fat accumulation induced by chemical agents [73, 74].

Through AMPK‐dependent inhibition of mTORC1 signalling (KER: KE1 ⟶ KE2), fasting also suppresses anabolic pathways such as protein and lipid biosynthesis (KE2), while releasing the autophagic machinery required for cellular recycling [37, 38]. This also induces “lipophagy”, the autophagic degradation of lipid droplets in hepatocytes (KE4) [75, 76]. In hepatocytes and mouse liver, autophagy inhibition increased triglyceride content ∼2‐fold, demonstrating direct lipid clearance via autophagic flux [77]. The resulting autophagy and lipophagy activation (KE4) further support hepatic energy balance by degrading intracellular components and providing substrates for oxidation (KER: KE2 ⟶ KE4) [78]. In parallel, AMPK activation suppresses hepatic lipogenesis (KER: KE1 ⟶ KE3), as fasting reduces insulin and nutrient signals that normally sustain SREBP‐1c–mediated de novo lipogenesis (KE3) [79, 80]. Glucocorticoid‐dependent induction of KLF15 and downregulation of SREBP‐1c reinforce this suppression, ensuring that lipid synthesis ceases while oxidation dominates [81].

The combination of autophagy‐derived substrates and AMPK/PPARα co‐activation drives fatty acid oxidation (KER: KE1 ⟶ KE4) [82] and ketogenesis (KE5). As fasting progresses, adipose tissue lipolysis releases FFAs into circulation, which are taken up by the liver as an alternate fuel. In humans, plasma ketones begin to rise within 24 h of fasting [46].

In parallel, fasting generates immunometabolic signals (KE8) that may attenuate hepatic inflammation and immune recruitment. The acute energy deficit in hepatocytes (MIE) lowers systemic CCL2 signalling via AMPK activation which results in a reduction of monocyte release from the bone marrow and thereby decreases inflammatory immune tone (KE9) without impairing emergency immune responses (KER: KE1 ⟶ KE8) [83]. Studies of IF suggest lower baseline circulating proinflammatory cytokines [84]. This can further be mediated by β‐hydroxybutyrate which can also act as a signalling metabolite, with reported inhibition of NLRP3 inflammasome–mediated IL‐1β and IL‐18 production in monocytes/macrophages (KER: KE5 ⟶ KE8) [85]. In a mouse model, IF also improved liver fat and insulin resistance with these benefits linked to macrophage migration inhibitory factor [86], an immunoregulatory cytokine well characterised for its antifibrotic effects [87]. This is confirmed in experimental NASH, where a 5:2 IF regimen improved established inflammation and fibrosis and reduced progression toward liver cancer via AMPK/PPARα co‐activation [31].

Another mechanism involved in the resolution of MASLD is the mitigation of oxidative stress. In MASLD, excess lipid storage in the liver promotes oxidative stress by activating several ROS‐producing systems, notably mitochondria, the endoplasmic reticulum, and NADPH oxidase [88]. Elevated ROS then interferes with insulin signalling and reshapes the regulation of lipid‐handling enzymes, which can further sustain steatosis and metabolic dysfunction. In this context, extended fasting has been reported to increase overall antioxidant capacity and lower lipid peroxidation (KE11), an effect that may be partly explained by a rise in circulating uric acid [89]. Ketosis from fasting increases uric acid because ketone bodies compete with uric acid for excretion in the kidneys (KER: KE5 ⟶ KE10), leading to higher blood levels [90]. Uric acid is a strong antioxidant in plasma [91], and experimental suppression of uric acid production with allopurinol has been associated with poorer physical performance and higher oxidative stress [92], supporting the relevance of this protective mechanism.

As hepatic lipid stores are mobilised and oxidised, hepatic and systemic insulin sensitivity improve (KER: KE5 ⟶ KE6) [93], leading to attenuation of insulin‐stimulated lipogenesis (KE6) [94]. With hepatic lipid overload reduced, insulin signalling normalises, hyperinsulinaemia abates, and inappropriate gluconeogenesis and triglyceride synthesis decline, as seen in the case of chronic DPP‐4 inhibition with linagliptin [95]. The reduction in oxidative stress further improves insulin sensitivity [96]. Ultimately, the improved insulin sensitivity sustains hepatic fat reduction, closing a positive feedback loop that consolidates the BO: resolution of MASLD.

## 7. Discussion: Limitations and Considerations in Applying the BOP Framework to Fasting

While BOPs offer a systematic approach to organise mechanistic knowledge on fasting, the framework also has limitations that warrant careful consideration. Unlike pharmacological exposures, fasting is not a unitary intervention but a family of practices, ranging from water‐only abstinence to FMDs, IF and very‐low‐calorie regimens, that differ substantially in duration, macronutrient composition, and energy load. These variations complicate the specification of a single MIE. For instance, a water‐only fast leads to rapid glycogen depletion and ketosis within 24–48 h, whereas a modified fast with partial caloric intake may produce only mild ketosis depending on protein and carbohydrate content. Current BOPs risk oversimplification if they assume a uniform initiating event without addressing these contextual differences.

### 7.1. Role of Adjunctive Lifestyle Therapies

Adjunctive lifestyle therapies represent another source of complexity. In clinical and wellness contexts, fasting is often combined with exercise, yoga, meditation or other behavioural interventions that independently influence metabolism, immunity and psychological state. Physical activity, in particular, interacts with fasting by enhancing fatty acid oxidation, preserving lean mass and modulating insulin sensitivity. Whether these effects should be modelled as parallel pathways or as modulators of fasting‐induced KEs is an open methodological question. Ignoring such co‐interventions could misattribute outcomes to fasting alone and weaken the causal specificity of the pathway.

### 7.2. Taking Into Account the Food Reintroduction

Heterogeneity in food reintroduction is an additional limitation. Many of the sustained benefits attributed to fasting depend on dietary quality after the fasting period. For example, the anti‐inflammatory state induced during fasting can be reinforced by a Mediterranean or plant‐forward diet but quickly reversed by reintroduction of ultra‐processed or high‐sugar foods [97]. Similarly, improvements in hepatic steatosis or insulin resistance may be lost if postfasting nutrition drives lipogenesis. Studies in mice suggest that low‐carbohydrate or ketogenic food reintroduction could keep the body in a fat‐adapted state longer, potentially maintaining some anti‐inflammatory and ketotic benefits [98]. In a case report of a 57‐year‐old man with poorly controlled Type 2 diabetes, repeated medically supervised LTFs (11–20 days) followed by a gradual reintroduction of food using a hypocaloric lacto‐vegetarian diet (800–1800 kcal/day) allowed sustained weight loss and improved glycaemic control suggesting that fasting may act as a trigger for durable metabolic reprogramming when it is coupled to sustained lifestyle changes [99].

This phase of food reintroduction should also be medically supervised because it also has safety implications: while refeeding syndrome is unlikely in typical populations undergoing medically supervised fasting with gradual food reintroduction, because it only occurs in cases of malnourishment, insulin‐driven shifts in phosphate, potassium, and magnesium remain a theoretical risk in vulnerable individuals [100].

Because the BOP framework typically terminates at a BO, it risks neglecting the temporal fragility of these improvements. Explicitly modelling food reintroduction as a late‐stage modulator may be essential to accurately reflect real‐world outcomes.

### 7.3. Applicability Domains: Who Benefits, Who is at Risk

Population diversity also presents limitations. Most mechanistic studies on fasting have been performed in relatively healthy or overweight adults, with fewer data in elderly populations, individuals with multimorbidity, or those on medications that alter metabolism. Sex differences in lipid handling and ketone dynamics, age‐related declines in mitochondrial and immune function, and ethnic differences in metabolic risk profiles all influence pathway strength. BOPs constructed from homogeneous cohorts may not apply to broader clinical populations without explicit specification of these boundaries.

Recurrent IF can induce adaptive changes that modify the hepatic response to subsequent fasting bouts. In mice exposed to ADF [101], hepatocytes showed a “sensitised” transcriptional programme during a later fast, with stronger induction of ketogenesis‐related genes and increased enhancer activity. This was associated with greater PPARα binding and translated into augmented ketone production compared with a first fasting bout.

A further consideration is safety and adverse events. While the BOP framework is designed to map BOs, fasting can also trigger negative effects, particularly when unsupervised or in vulnerable individuals. Hypotension, hypoglycaemia, electrolyte imbalance and exacerbation of eating disorders are recognised risks.

### 7.4. Need for Quantification and Stronger Causal Validation

In addition, quantification is essential. Even in lifestyle interventions, many nodes can be associated with measurable thresholds, such as changes in metabolite concentrations. Incorporating quantitative features into BOPs aligns with ongoing efforts to develop quantitative AOPs (qAOPs), where dose–response and temporal relationships are used to predict the likelihood of outcomes [102].

Dose in fasting is defined not only by duration but also by the depth of energy deficit, macronutrient composition, and repetition over time. In our large 2019 observational study of periodic fasting (mean 8.5 days, range 6–38), the reduction in fatty liver index showed a modest but significant dose–response relationship with fasting duration (correlation with fasting days), and it also tracked with the magnitude of BMI reduction, supporting the idea that fasting ‘dose’ reflects both time and the depth of the energy deficit [19].

Taken together, these considerations suggest that while BOPs are a powerful organising tool, their application to fasting requires careful attention to intervention heterogeneity, dose–response, adjunct therapies, refeeding, population diversity, safety, and uneven evidence strength. Addressing these challenges will refine the framework and enhance its value for guiding research, clinical practice, and policy.

## Funding

The study was financed by Buchinger Wilhelmi Development & Holding GmbH.

## Disclosure

The funder had no impact on the outcomes of the study.

## Conflicts of Interest

Robin Mesnage and Verena Buchinger are employees of the Buchinger Wilhelmi Development and Holding GmbH.

## Data Availability

Data sharing is not applicable to this article as no datasets were generated or analysed during the current study.
